# Task-Specific Motor Rehabilitation Therapy After Stroke Improves Performance in a Different Motor Task: Translational Evidence

**DOI:** 10.1007/s12975-016-0519-x

**Published:** 2017-01-14

**Authors:** M. El Amki, P. Baumgartner, O. Bracko, A. R. Luft, S Wegener

**Affiliations:** 1 Division of Vascular Neurology and Rehabilitation, Department of Neurology, University Hospital Zurich, University of Zurich, Frauenklinikstrasse 26, 8091 Zurich, Switzerland; 2 cereneo Center for Neurology and Rehabilitation, Vitznau, Switzerland

**Keywords:** Middle cerebral artery occlusion, Rehabilitation, Motor recovery, Stroke, Rats

## Abstract

While the stroke survivor with a motor deficit strives for recovery of all aspects of daily life movements, neurorehabilitation training is often task specific and does not generalize to movements other than the ones trained. In rodent models of post-stroke recovery, this problem is poorly investigated as the training task is often the same as the one that measures motor function. The present study investigated whether motor training by pellet reaching translates into enhancement of different motor functions in rats after stroke. Adult rats were subjected to 60-min middle cerebral artery occlusion (MCAO). Five days after stroke, animals received either training consisting of 7 days of pellet reaching with the affected forelimb (*n* = 18) or no training (*n* = 18). Sensorimotor deficits were assessed using the sticky tape test and a composite neuroscore. Infarct volumes were measured by T2-weighted MRI on day 28. Both groups of rats showed similar lesion volume and forelimb impairment after stroke. Trained animals improved in the sticky tape test after day 7 post-stroke reaching peak performance on day 14. More reaching attempts during rehabilitation were associated with a better performance in the sticky tape removal time. Task-oriented motor training generalizes to other motor functions after experimental stroke. Training intensity correlates with recovery.

**Electronic supplementary material:**

The online version of this article (doi:10.1007/s12975-016-0519-x) contains supplementary material, which is available to authorized users.

## Introduction

About 60% of stroke survivors suffer from motor disability 6 months after stroke [1, 2]. By training of motor skills, rehabilitation aims to maximize patients’ functional independence and quality of life. The physiological mechanisms of training interventions are incompletely understood, especially their generalization, i.e., how and how much improvement in the specific task trained generalizes to other movements. These mechanisms need to be explored in animal models to optimize and develop treatments.

In rodents, post-stroke motor rehabilitation by pellet-reaching training improves pellet-reaching success [3]. This is accompanied by reorganization in motor cortex regions controlling the affected limb [4], e.g., an increase in dendritic complexity [5, 6]. The issue of generalization of trained to other tasks has not been addressed in animal models of post-stroke recovery.

The present study investigated whether motor training by pellet reaching translates into improvement in other motor tasks in a rat stroke model. The transient middle cerebral artery occlusion (MCAO) was chosen for stroke induction, because the lesion is not confined to the motor cortex but has a variable spread towards adjacent cortical and subcortical areas, similar to human stroke.

## Materials and Methods

**Fig. 1:**
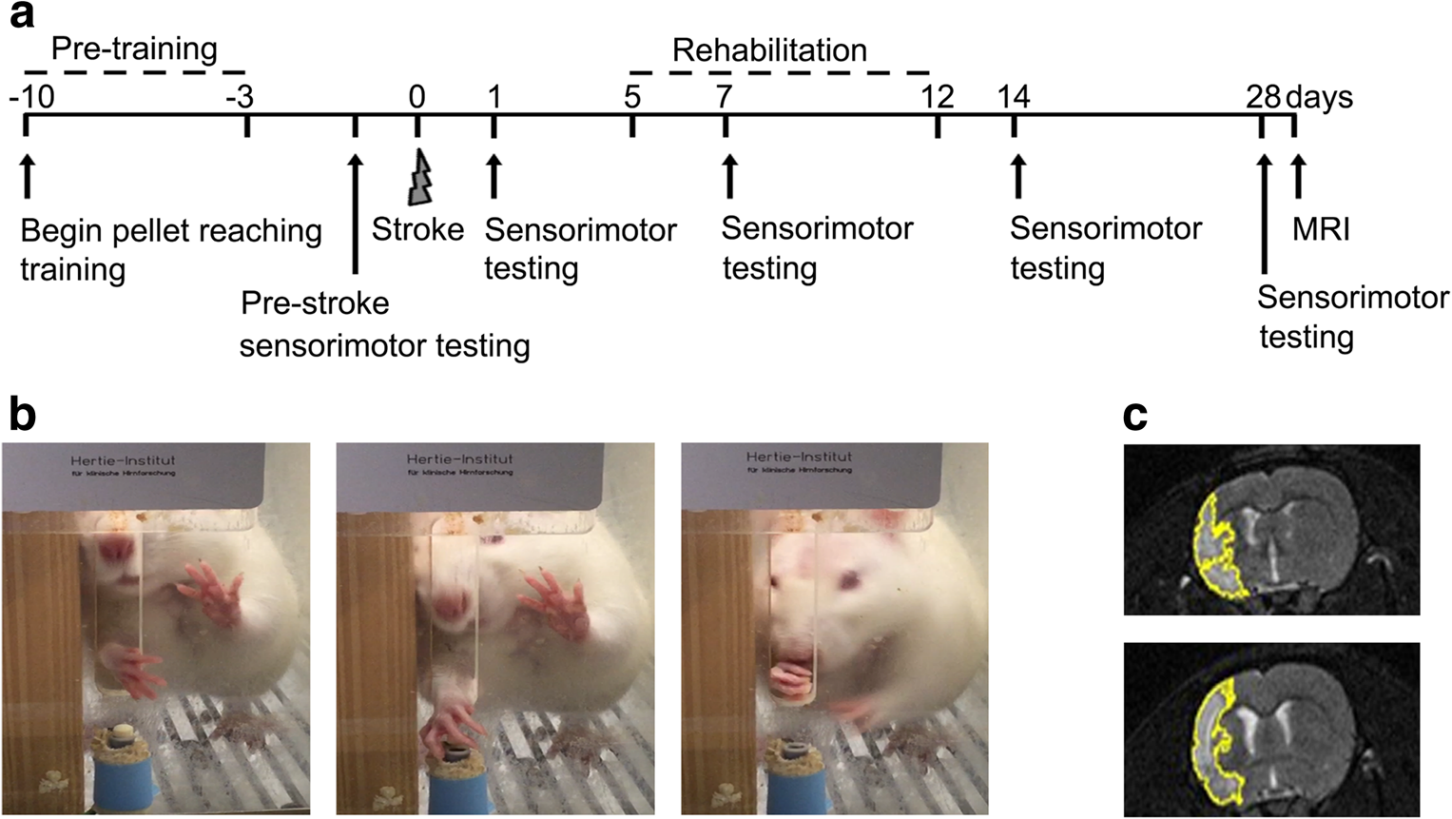
Flow of the experiments. **a** Experimental schedule. **b** Photos illustrating rats during pellet-reaching training. **c** Representative MRI-T2 images from the rehabilitation and no rehabilitation group 28 days after MCAO

All experiments were performed in accordance with the guidelines and regulations approved by the Federal Veterinary Office of Switzerland (Veterinary Office of the Canton of Zurich). Adult male Sprague Dawley rats (280 to 310 mg body weight) were used. The experimental setup is shown in Fig. 1. Out of 46 animals, 5 animals died or had to be euthanized prematurely due to massive infarction. Five rats were excluded because of insufficient stroke induction as judged by less than 15 s to remove the sticky tape on day 1 after MCAO. Analysis of sensorimotor scores and stroke lesion size was performed by an investigator blinded to group assignment.

### Middle Cerebral Artery Occlusion

During surgery, rats were anesthetized using facemask inhalation of 1.5 to 2.5% isoflurane in a 2:1 N_2_O:O_2_ atmosphere. Animals were subjected to 60-min MCAO as described previously [7]. Laser Doppler flowmetry (Moor Instruments Ltd., Millwey, UK) was used to confirm the occlusion of the MCA. The probe was fixed on the skull in the left MCA territory and rats with less than 30% drop in cerebral blood flow were excluded from the studies. The body temperature was monitored throughout surgery using a rectal probe and maintained at 37 °C with a normothermic blanket (Harvard Apparatus, Edenbridge, Kent, UK).

### Pre-training, Motor Skill Learning, and Motor Rehabilitation

Training procedures were conducted as previously described [8]. More details are available as supplemental data. One daily session consisting of 100 trials or a maximum time of 45 min was performed for each animal. Reaching performance was measured by counting the number of successful reaches.

Animals were randomized into the rehabilitation (“rehab”) or no rehabilitation (“no rehab”) group on day 4 after MCAO. Five days after MCAO (D5), animals of the rehab group received daily pellet-reaching sessions for 7 days until D12. The number of successful reaches was tested in all animals at D5 and D12 after MCAO.

### Sensorimotor Testing

Sensorimotor function was evaluated using the adhesive tape removal test as well as a composite observational neurological score (see supplemental methods) [7].

### Magnetic Resonance Imaging Methods

On day 28 after MCAO, magnetic resonance imaging (MRI) was carried out on a 4.7-T rodent scanner (see also supplemental material).

## Statistical Analysis

A sample size calculation (alpha 0.05, power 0.8) was performed based on previous sticky tape removal test data in rats after MCAO. We calculated a minimum sample size of 16 animals per group to show an effect size of 0.9. Power calculation was performed using G Power Software (version 3.1.5). Statistical analyses were done using SPSS v12.0 for Windows. All values are given as mean ± standard error of mean (s.e.m.). For group comparisons, either the two-sided independent sample *t* test or the nonparametric Mann-Whitney *U* test was used depending on data distribution. A *p* value <0.05 was considered significant.

## Results

**Fig. 2:**
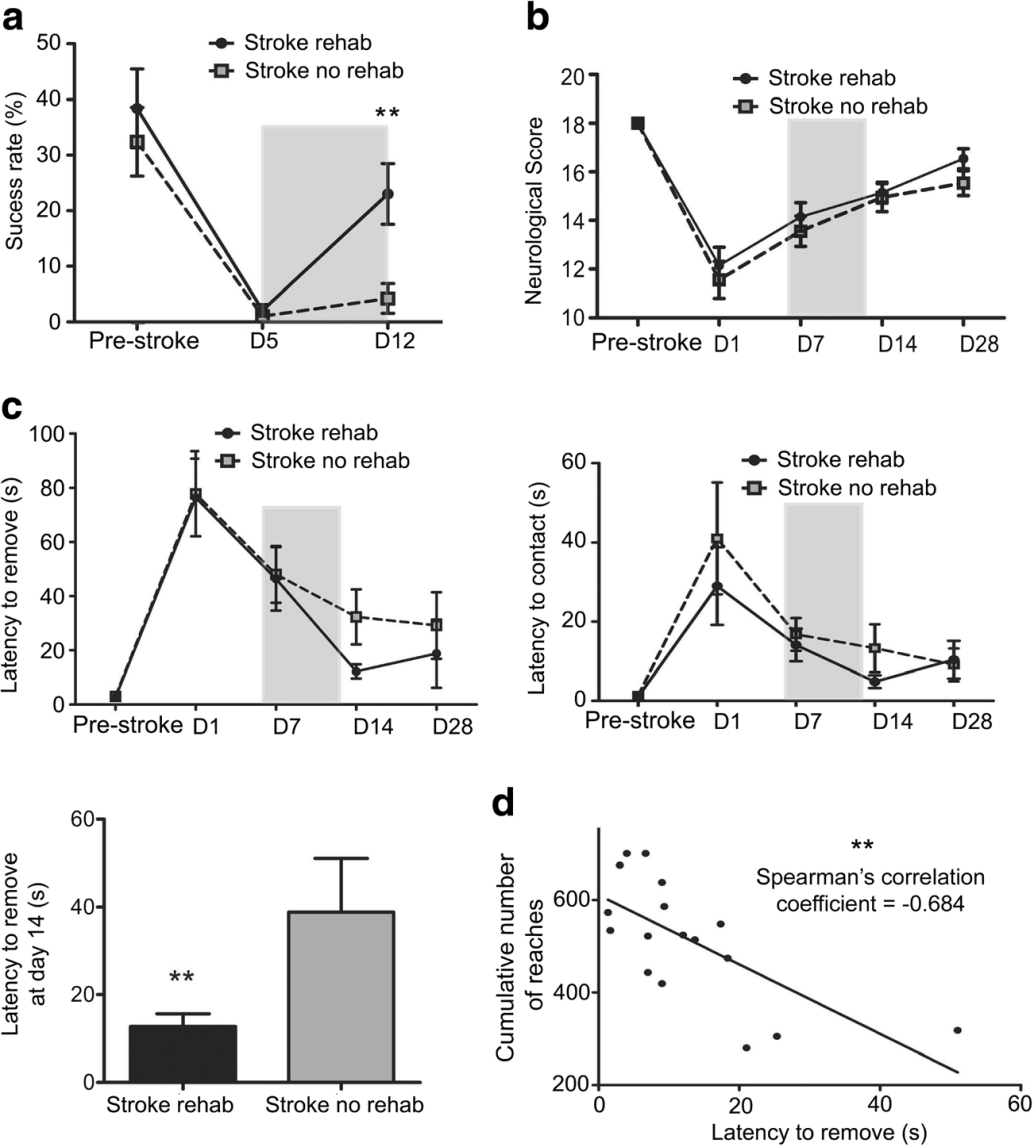
Pellet-reaching training improved motor function after MCAO. **a** Success rate for reaching task of rehab (*n* = 18) and no rehab rats (*n* = 18) from pre-stroke to D12. **b** Composite neurological score. **c** Sticky tape test: latency to remove (*left*) and contact (*right*) a sticky tape applied to the right forepaw on all time points tested. The *gray*-*shaded box* in **a**–**c** indicates duration of rehabilitation. No repeated measures ANOVA was performed because of the short (7d) intervention period. *Lower left*: latency to remove sticky tape at D14 in rehab versus no rehab rats. **d** Correlation between cumulative number of pellet reaches and latency to remove the sticky tape at D14 in rehab rats

Before stroke surgery, both groups achieved a similar reaching performance (38.1 ± 7.1% success rate for rehab versus 32.5 ± 6.2% for no rehab, Fig. 2a). Five days after stroke, all animals had considerable problems in obtaining pellets with the impaired limb (2.1 ± 1.0% in rehab and 2.0 ± 0.9% in no rehab group). As expected, animals that received daily rehabilitation from D5 to D12 after MCAO achieved a higher pellet-reaching success rate at D12 (23.0 ± 5.5% versus 4.2 ± 2.6% *p* < 0.01; Fig. 2a). MRI stroke lesion analysis showed no difference between the two groups (165 ± 41.9 mm^3^ in rehab versus 176 ± 38.7 mm^3^ in no rehab animals). In the composite neurological score, trained and control animals where similarly affected after stroke (Fig. 2b). In the sticky tape test, deficits to perceive and remove the tape on the right side were noted in all animals at D1 after MCAO (Fig. 2c). However, animals with rehabilitation performed faster in the sticky tape removal task (motor component) on D14 than animals without rehabilitation (12 ± 2.6 s versus 38 ± 10.2 s; *p* = 0.007). A high-cumulative number of pellet reaches during training was negatively correlated with the time to remove the sticky tape on D14 (*R* = −0.68, *p* < 0.01, Fig. 2d).

## Discussion

Although intense rehabilitation incorporating exercise, forelimb constraint therapy and task-specific pellet reaching have been shown to enhance performance in a similar but not identical task-specific test (tray-reaching) in rats after brain injury, our study demonstrates that a task-oriented motor rehabilitation algorithm alone can improve motor function in a task that is substantially different from the trained one [9]. Daily pellet-reaching training enhanced motor ability for sticky tape removal.

Motor rehabilitation by pellet-reaching training is focused on recovery of highly specific functions (skilled grasping ability) and requires intensive training and practice of the impaired function [10]. As potential mechanisms mediating the beneficial effects of motor rehabilitation, pro-plastic and neurotrophic factors such as brain-derived neurotrophic factor (BDNF) and insulin-like growth factor (IGF-1) have been indicated [11]. The control group did not show significant recovery in the skilled reaching task. Neither the sensory component of the sticky tape test nor the composite neurologic score was influenced by motor rehabilitation in our model. This argues for specific effects on motor recovery and against other functional domains, such as sensation or neglect involved in the observed regain of function.

The improvement in sticky tape removal was correlated with more successful pellet-reaching attempts, indicating that a higher intensity of training may be beneficial. Since the effect was most pronounced on day 14 and less evident on day 28, our experimental data argue for continued motor rehabilitation for stroke patients in order to maintain a sustained effect on motor function.

## Summary

Although studies generally support the concept that motor rehabilitation is associated with improved outcomes after stroke, few experimental studies have specifically tested whether focused rehabilitation improves other motor outcomes. To our knowledge, our study represents the first translational evidence that task-specific training after stroke generalizes to a different motor task.

## Electronic Supplementary Material

ESM 1 (DOCX 15 kb).

## References

[1] Claflin ES, Krishnan C, Khot SP (2015). Emerging treatments for motor rehabilitation after stroke. Neurohospitalist.

[2] Winstein CJ, Wolf SL, Dromerick AW, Lane CJ, Nelsen MA, Lewthwaite R, Cen SY, Azen SP (2016). Interdisciplinary comprehensive arm rehabilitation evaluation (ICARE) investigative team. Effect of a task-oriented rehabilitation program on upper extremity recovery following motor stroke: the ICARE randomized clinical trial. JAMA.

[3] Alaverdashvili M, Whishaw IQ (2010). Compensation aids skilled reaching in aging and in recovery from forelimb motor cortex stroke in the rat. Neuroscience.

[4] Clarkson AN, López-Valdés HE, Overman JJ, Charles AC, Brennan KC, Carmichael ST (2013). Multimodal examination of structural and functional remapping in the mouse photothrombotic stroke model. J Cereb Blood Flow Metab.

[5] Jones TA, Schallert T (1994). Use-dependent growth of pyramidal neurons after neocortical damage. J Neurosci.

[6] Chu CJ, Jones TA (2000). Experience-dependent structural plasticity in cortex heterotopic to focal sensorimotor cortical damage. Exp Neurol.

[7] Bracko O, Di Pietro V, Lazzarino G, Amorini AM, Tavazzi B, Artmann J, Wong EC, Buxton RB, Weller M, Luft AR, Wegener S (2014). 3-Nitropropionic acid-induced ischemia tolerance in the rat brain is mediated by reduced metabolic activity and cerebral blood flow. J Cereb Blood Flow Metab.

[8] Rioult-Pedotti M-S, Pekanovic A, Atiemo CO, Marshall J, Luft AR. Dopamine promotes motor cortex plasticity and motor skill learning via PLC activation. PLoS One 2015; 10. 10.1371/journal.pone.0124986 PMC4418826 25938462

[9] Combs HL, Jones TA, Kozlowski DA, Adkins DL (2016). Combinatorial motor training results in functional reorganization of remaining motor cortex after controlled cortical impact in rats. J Neurotrauma.

[10] Wang L, Conner JM, Nagahara AH, Tuszynski MH (2016). Rehabilitation drives enhancement of neuronal structure in functionally relevant neuronal subsets. Proc Natl Acad Sci U S A.

[11] Ploughman M, Austin MW, Glynn L, Corbett D (2015). The effects of poststroke aerobic exercise on neuroplasticity: a systematic review of animal and clinical studies. Transl Stroke Res.

