# Supplementary material for: Task-Specific Motor Rehabilitation Therapy After Stroke Improves Performance in a Different Motor Task: Translational Evidence
Source: Transl Stroke Res. 2017 Jan 14;8(4):347–50. doi: 10.1007/s12975-016-0519-x (PMC5493722; doi:10.1007/s12975-016-0519-x)
Supplement: Supplementary file 1 — (DOCX 15 kb) [file 12975_2016_519_MOESM1_ESM.docx]

**Supplemental Material**

**Pre-training, motor skill learning and motor rehabilitation**

Briefly, rats were trained to reach and grasp for a food pellet placed outside the home cage at a specified distance. To gain access to the pellet, the animal had to learn to open a motorized sliding door by nose poking to an inductive sensor. After becoming familiar with cage and food pellets, pre-training with the pellet accessible by tongue (2-3 days) was performed to teach animals how to open the door (concept) and where the pellet was. Motor skill learning was subsequently initiated by moving the pellet to the left side, requiring the rat to use the right (later affected) forelimb to retrieve the pellet. After 5 to 8 days of motor skill training, the plateau performance was reached. At this stage, rats were able to perform 100 trials in 10 minutes with an average success rate of 30 - 40%. At the end of pre-training, animals that successfully performed 100 trials in less than 10 minutes and had >25 successful reaches were included. For rehabilitation, animals were placed in the cage without any additional handling and did either 100 reaching trials or, if this was not achieved, 45 min of training in the cage.

**Sensorimotor testing**

For the sticky tape test, two strips of tape (18 x 12mm) were applied to both forepaws in random order. The time the animals took to contact (sensory function/neglect) and remove (motor function) the tape on both sides (left and right) was recorded. Before stroke, animals were trained to remove both tapes within 10 seconds (3 sessions). During the experiment, results from two trials at least 3 minutes apart were averaged at each session.

The 18-point composite neurological score incorporates the observation of (1) spontaneous activity, (2) symmetry in limb movement, (3) forepaw outstretching, (4) climbing, (5) body proprioception, and (6) response to vibrissae touch, so that 18 points indicate no neurologic deficit^7^.

**Magnetic Resonance Imaging Methods (MRI)**

On day 28 after MCAO, MRI was carried out at the Biology core laboratory at the University Zurich on a rodent 4.7 T MRI-system, with a receive-only surface coil. Animals were anesthetized with isoflurane (2-2.5%) and placed in a custom-built cradle. T2-weighted MRI sequences were obtained using a turbo spin echo sequence with RARE-factor = 4, effective echo time (TE) = 50 ms, repetition time (TR) = 2.9 s, 5 averages. Body temperature and respiratory rate were continuously monitored. Images were analyzed using Image J software (NIH) by semi-automatically delineating the area of T2-hyperintensity with a thresholding tool.
